# Spontaneous Coronary Artery Dissection: An Updated Comprehensive Review

**DOI:** 10.7759/cureus.55106

**Published:** 2024-02-27

**Authors:** Chibuike C Agwuegbo, Eman N Ahmed, Emmanuel Olumuyide, Serin Moideen Sheriff, Sahani A Waduge

**Affiliations:** 1 Internal Medicine, Southwest Healthcare, Temecula, USA; 2 Internal Medicine, Alfaisal University College of Medicine, Riyadh, SAU; 3 Internal Medicine, Insight Hospital and Medical Center, Chicago, USA; 4 Cardiovascular Medicine, Mayo Clinic, Jacksonville, USA; 5 Internal Medicine, University of Science and Technology, Chittagong, BGD

**Keywords:** spontaneous, myocardial infarction, coronary angiography, dissection, acute coronary syndrome, coronary artery dissection, scad

## Abstract

Spontaneous coronary artery dissection (SCAD) is defined as a non-iatrogenic, non-traumatic separation of the coronary artery wall, which has gained considerable recognition as an important cause of acute coronary syndrome. Despite the emerging evidence, it is still frequently missed and requires a high index of suspicion, as failure to accurately identify SCAD promptly could prove fatal. SCAD is most prevalent among middle-aged women, although it can also be found in men and postmenopausal women. Risk factors of SCAD include exogenous hormone use, physical and emotional stressors, pregnancy, and several inflammatory and connective tissue disorders. COVID-19 also contributes to the prevalence of SCAD. SCAD is classified into four main types based on the angiographic findings - type 1, type 2, type 3, and type 4. The gold standard for diagnosis is coronary angiography; however, intracardiac imaging is useful if diagnostic doubts persist. Despite the increasing recognition of this disease, there is a paucity in the guidelines on the management of SCAD. Management may be conservative, medical, or interventional. Cardiac rehabilitation is also necessary in the management of patients with SCAD. In light of the gaps in evidence, the authors aim to provide a comprehensive review of the existing literature, outlining the pathophysiology, classification, and, most importantly, the evidence and pitfalls circulating diagnosis, acute, and long-term management of SCAD.

## Introduction and background

Spontaneous coronary artery dissection (SCAD) is defined as a non-iatrogenic, non-traumatic separation of the coronary artery wall, compromising coronary circulation and perfusion ultimately causing myocardial ischemia and acute coronary syndrome (ACS). SCAD is frequently misdiagnosed as it requires a high index of suspicion for accurate identification in a timely manner. Failure to do so could prove fatal due to the progressive nature of the disease and inappropriate management [1].

The clinical presentation of SCAD is similar to atherosclerotic ACS; therefore, it is often misdiagnosed as such. This is particularly dangerous as the treatment modalities, although similar, could lead to more devastating complications, especially in the setting of percutaneous coronary interventions in SCAD cases, which could cause further propagation of the dissection, leading to significant morbidity and mortality [1,2].

Despite the increasing recognition of SCAD, there is still a paucity of literature on the diagnosis and management of SCAD. We aim to discuss current literature on the epidemiology, risk factors, proposed mechanisms, classification, clinical presentations, diagnostic modalities, and the current management recommendations for SCAD, with a glimpse into the future trends.

## Review

In 1931, an aggressive retching episode that led to SCAD has been documented [3]. It is an often-undiagnosed disease, previously only diagnosed during postmortem care [1]. Since then, the recognition and understanding of the etiologies, pathogenesis, and diagnosis of SCAD has gradually increased over the years. Despite the increasing recognition of SCAD as an important cause of ACS, there is still a paucity of clear management guidelines specifically for SCAD [4]. Hence, clinicians have relied on guidelines for the management of ACS in the treatment of SCAD, which has been less than ideal as these guidelines were designed for atherosclerotic causes of ACS [5].

Epidemiology

The incidence and the true prevalence of SCAD remain uncertain because it is frequently undiagnosed, but studies have shown the estimated prevalence of SCAD is as high as 2.1% of all patients presenting with ACS [6]. It is most commonly seen in healthy younger women (87%-95% of SCAD) with a mean age of presentation between 44 and 55 years [2,6,7]. SCAD most commonly occurs in patients with few or no traditional cardiovascular risk factors [8,9]. It is the most common cause of myocardial infarction (MI) in pregnant women with a prevalence of up to 43% [10]. Although data regarding the prevalence of SCAD in men are rare, we previously reported a case depicting SCAD in a young male with no traditional risk factors [11]. The age at presentation in men is lower (mean age of 48 years) when compared with women (mean age of 52 years) [12]. The left anterior descending (LAD) artery is most commonly affected in patients, with a prevalence of about 50%, followed by the left circumflex artery, right coronary artery, and left main artery [13]. Multivessel SCAD occurs in 9-23% of cases [8,14]. The mid to distal segments are the most commonly affected segments; the proximal segments are involved in less than 10% of the cases [5,7].

Risk factors

There is an influence by a combination of genetic, hormonal, and environmental factors on SCAD. The exact causes are not well-known, but studies have shown that extreme emotional upset, extreme physical activity, pregnancy or postpartum, fibromuscular dysplasia, and connective tissue disorders such as Ehlers-Danlos syndrome, Marfan syndrome, and cigarette smoking are well-documented risk factors of SCAD [5-7,15]. Among precipitants, emotional stressors appeared more commonly in women, whereas physical stressors were seen in men. Some unusual precipitating factors, such as cocaine exposure, severe retching, Valsalva straining, and certain medications, have also been reported [5,7,10]. It is important to note that SCAD can also occur in individuals with no identifiable traditional cardiovascular risk factors such as high cholesterol and diabetes [15,16].

Pregnancy: SCAD is the leading cause of MI in pregnant women [10]. Female sex hormones play an important role in the pathogenesis of SCAD, as estrogen has been known to affect the microvasculature and reactivity of coronary vasculature [17]. The hemodynamic changes that occur during pregnancy due to the surge of female sex hormones, as well as the increased shear stress on the vessel walls, increase the risk of SCAD. Risk factors that increase SCAD incidence in pregnancy include multiple previous pregnancies, in-vitro fertilization, pre-eclampsia, and a prior history of hypertension and hyperlipidemia [17].

Arteriopathies and inflammatory conditions: Fibromuscular dysplasia is the disease most frequently associated with SCAD, accounting for up to 72% of SCAD cases [9,18]. This is due to the pathological changes in the arteries such as stenosis and aneurysms, which make them more susceptible to dissection. Connective tissue diseases, such as Marfan’s syndrome, Ehlers-Danlos syndrome, Loeys-Dietz syndrome, and autosomal dominant polycystic kidney disease, are responsible for < 5% of SCAD patients [19,20]. A study by Krittanawong et al. noted a prevalence of SCAD in up to 0.42% of patients with systemic lupus erythematosus [21].

Exogenous hormone use: Hormonal replacement therapy in menopausal women increases risks of SCAD due to increased oxidative stress, hypercoagulability, arrhythmogenesis, and vasomotor reactivity. Both combined oral estrogen and progesterone and estrogen-only therapies have been associated with an increased risk of SCAD [22-24]. Anabolic steroid use has also been reported in patients with SCAD, likely due to increased weakness of the vessel wall, leading to hypercoagulability and platelet activation and aggregation [23].

Physical and emotional stressors: Sudden or severe stress, including exercise, Valsalva maneuvers, coughing bouts or retching/vomiting, have been implicated as significant risk factors of SCAD. In fact, the very first case of SCAD ever described was in a patient who presented after severe retching and vomiting [3]. Saw et al. reported that physical and emotional stressors are responsible for up to 56.5% of SCAD patients [9]. These stressors cause a rise in catecholamine levels, thereby increasing coronary artery shear stress leading to SCAD. Amphetamine use and cocaine also increase the risk of SCAD due to similar mechanisms [5].

Genetics: Hayes et al. reported several genetic abnormalities predisposing to SCAD [5]. The genes associated with the risk for SCAD include F11R (the gene responsible for F11 receptor that regulates tight junction assembly), TLN1 (the gene encoding Talin 1, responsible for linking the actin cytoskeleton to the extracellular matrix), TSR1 (the gene which influences ribosome maturation factor and RNA formation), PHACTR1 (the gene encoding cytoskeleton actin), and EDN1 (encoding endothelin 1, which is a circulating vasoactive peptide) [5]. However, genetic testing for SCAD is not routinely done but may be considered in situations where diagnostic doubt persists.

COVID-19: Cytokine storm and inflammatory cascade caused by SARS-CoV-2 infection results in vascular compromise by increased endothelial dysfunction, thrombosis, and vascular spasm. Papageorgiou et al. conducted a systematic review of 16 cases of SCAD in patients with COVID-19 infections, reporting no mortality and good prognosis in all reported cases [25].

Pathogenesis

**Figure 1:**
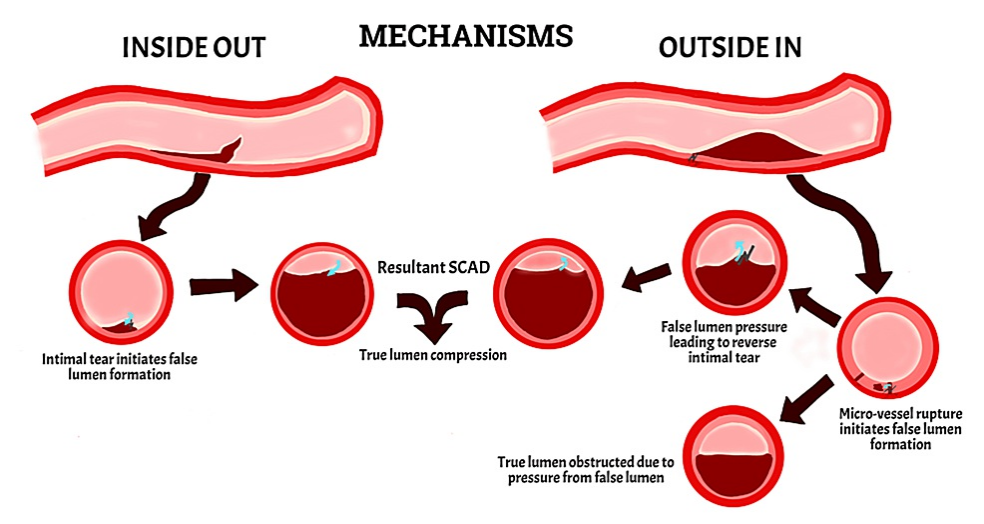
Illustration depicting the proposed mechanisms of pathogenesis of spontaneous coronary artery dissection (i.e., "Inside Out" and "Outside In" phenomena. The blue arrows represent the direction of blood flow facilitating false lumen formation (maroon) and resulting from an intimal insult (black bolt arrows) on the true lumen (beige). SCAD: Spontaneous Coronary Artery Dissection Image Credits: Eman N. Ahmed

The pathogenesis of SCAD is not fully understood, but it has been suggested that SCAD occurs from a tear in the coronary arteries, causing reduced blood flow to the heart. The development can be explained by two mechanisms: the "inside-out" phenomenon, where blood leaks from the inner layer of the artery to the middle layer, and the "outside-in" phenomenon, where small blood vessels within the middle layer rupture, leading to a buildup of blood within the arterial wall (Fig. 1). The first mechanism can be seen on angiography as multiple radiolucent lumens with slacking contrast in the vessel. The second mechanism presents as compression due to hematoma and needs imaging to differentiate from atherosclerotic stenosis [7]. Both mechanisms develop a false channel in the middle layer, which causes obstruction or compression of the vessel, leading to decreased blood flow to the heart and causing myocardial ischemia [1,2].

Classification of SCAD

**Figure 2:**
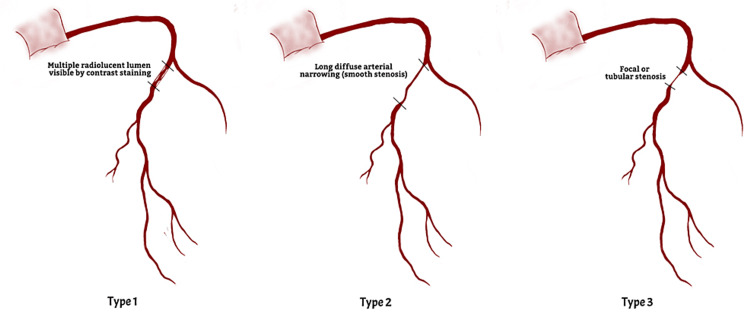
Illustration depicting major angiographic classification of spontaneous coronary artery dissection. The image shows the classic appearance of Type 1, 2, and 3 dissections in the mid-proximal portion of the left anterior descending artery Image credits: Eman N. Ahmed

Saw [26] proposed a classification for SCAD based on coronary angiography (Fig. 2). Table 1 describes the different features found in the different types of SCAD.

**Table 1:** Classification of SCAD. IVUS - Intravascular Ultrasound; OCT - Optical Coherence Tomography; SCAD - Spontaneous Coronary Artery Dissection

Type of SCAD	Differentiating Features
Type 1	Angiographic features include contrast dye staining the arterial wall and the presence of multiple radiolucent lumens. Found in 29-48% of cases
Type 2	The most common type of SCAD (~60-75%). Angiographic features include diffuse smooth stenosis of the arterial lumen with varying severity, intramural hematoma and double lumen visible on intracoronary imaging. Subtypes: Type 2A - Normal artery caliber proximal and distal to dissection. Type 2B - Dissection usually involves the mid-arterial segment. In severe cases, it can extend to the distal tip of the artery. Some type 2 SCAD (short length) may require intra-coronary imaging for diagnosis. Does not respond to intra-coronary nitrates
Intermediate Type 1/2	Contain angiographic features of both type 1 and 2 SCAD including arterial walls staining with multiple radiolucent lumens (type 1 SCAD mimic), and diffuse smooth stenosis (type 2 SCAD mimic)
Type 3	Often misdiagnosed since angiographic findings mimic atherosclerosis. It is found in 2-4% of cases. Angiographic features include hazy stenosis, long lesions (11-20 mm), and linear stenosis. They often require intra-coronary OCT/IVUS to confirm the presence of an intramural hematoma.
Type 4	Most commonly involves a distal segment of the artery. Angiographic features include sudden total coronary vascular occlusion

Clinical presentation

The clinical presentation of SCAD is indistinguishable from atherosclerotic ACS with symptoms, such as sweating, palpitations, dizziness, and shortness of breath. Patients usually present to the emergency department with chest pain as the most common symptom. In some patients, chest pain radiates to the shoulder, arm, back, or jaw. A smaller percentage of SCAD patients can present with complications such as ventricular arrhythmia, cardiogenic shock, or sudden cardiac arrest [5].

Diagnosis of SCAD

Diagnosis of SCAD relies heavily on clinical suspicion through history, physical, laboratory, and imaging investigations. Laboratory cardiac markers show elevated troponin levels, followed by ECG findings mostly consistent with non-ST/ST segment ACS, making the diagnosis of SCAD even more obscure. A meta-analysis of case series reported that SCAD presents as an ST-elevation MI pattern in 48% of patients, non-ST elevation MI in 36%, and unstable angina presentation in 6.5% [4,5,26].

The gold standard for diagnosis of SCAD and usually the only imaging modality necessary is coronary angiography. However, coronary angiography is limited by its two-dimensional nature, which does not adequately capture the arterial wall [5]. Additionally, the coronary anatomy in some cases of tortuous vessels or smaller lumen-size vessels may limit the safety of intracoronary imaging (ICI), necessitating the use of adjunct methods of imaging for diagnosis. This includes modalities such as cardiac magnetic resonance (CMR) and noninvasive coronary computed tomography angiography (CCTA) [5]. ICI modalities such as intravascular ultrasound (IVUS) and optical coherence tomography (OCT) provide visualization of arterial walls, including the intima, media, and adventitia. However, its use is limited due to cost, and in most cases, coronary angiography is sufficient to diagnose SCAD [27]. Therefore, the use of ICI such as IVUS and OCT is reserved for cases of persistent diagnostic doubt. 

Coronary angiography: Conventional angiographic findings of SCAD include the presence of multiple radiolucent lumens, radiolucent flaps, and extraluminal contrast staining, showing an intraluminal filling defect of a spiral dissection [27]. However, this traditional presentation has only been described in less than half of the SCAD cases. Other coronary angiography findings include small, long diffuse stenosis with varying severity and length, focal stenosis, and even a lack of intraluminal thrombus. The type of SCAD is further identified and classified based on the angiographic findings (Table 1). While coronary artery plaque rupture can be complicated by dissections, spontaneous dissections are more characterized by a long, smooth narrowing with distal tapering, with distal reconstitution within normal vessels extending into terminal branches [5].

IVUS: It has great depth penetration and offers the ability to detect false lumens, intimal tears, intraluminal thrombi, or hematomas. However, its use is limited by its poor spatial resolution of 150 µm, which confers an inability to identify all scattered abnormalities such as fenestrations and intimal-medial membranes [5]. Nevertheless, IVUS has wider availability and user-friendliness and does not require the use of contrast as compared to OCT.

OCT: It is a more advanced technology with a spatial resolution of 10-20 µm, which uses a light wave to delineate the lumen-intimal interface, providing longitudinal and transverse imaging images for visualizing the anatomy of vessels. Data collected by Jackson et al. on OCT showed that non-fenestrated thrombi resulted in increased false lumen pressure and true lumen compression compared to dissections with connected false-true lumens [28]. However, it is not without its pitfalls, including its need for IV contrast, which could potentially worsen the extension of SCAD by further extending the false lumen.

Coronary computed tomography angiography (CCTA): CCTA is more useful in low-to-intermediate risk patients [5]. Certain specifics have been identified distinguishing SCAD from atherosclerotic plaques on angiography such as comparative greater attenuation > 50 Hounsfield units, coronary tortuosity, and absent remodeling of the vessel [29]. Identifying features of CCTA in cases without intimal disruption is challenging and can be easily missed. Distal coronary arteries, or side branches, and smaller vessels are also easily missed on CCTA due to limitations in spatial and temporal resolution. Other pitfalls include motion artifacts, unknown specificity, and sensitivity, as well as low spatial resolution. CCTA is useful for noninvasive follow-up of patients with SCAD, especially in proximal and large arteries [5]. Pergola et al. recommend utilization of CCTA in acute settings, as well as for follow-up cases, suggesting a three to six months repeat imaging to observe for resolution [29]. On assessment of the accuracy of lesion resolution in a blinded study, Wong et al. noted a sensitivity of 72% and specificity of 53.8% by CCTA [30].

Management strategies

The demand to manage SCAD emerged as authors noted an increased prevalence in patients referred for coronary angiography as early as 1998 [31]. Additionally, developing up-to-date ACS invasive treatment guidelines and augmented usage of ICI have contributed to its increasing prevalence [32]. Managing SCAD is challenging due to the difference in response to treatment from ACS and the limited evidence from randomized trials. In the acute setting, SCAD is managed analogous to ACS treatment modalities, with medical and invasive therapies where interventionalists aim to restore flow distal to the lesion [5]. Experts help determine the treatment approach for these patients through high-risk and low-risk features. Thrombolysis in myocardial infarction (TIMI) flow 0-1, active ischemia, left main stenosis, and clinical instability are high-risk features justifying revascularization, while low-risk features such as hemodynamic stability and TIMI flow 2-3 can be treated with conservative approach [33]. They eventually require observation at cardiac rehabilitation (CR) centers as part of post-SCAD management.

Medical Modalities

As there are no clinical trials that standardize pharmacotherapy in SCAD survivors yet, the optimal medical intervention is still unclear, based on case observation, clinical experience, and extrapolation from non-SCAD ACS guidelines [34,35]. An analysis performed by Van Damme et al. on the use and frequency of medical therapy reported aspirin and P2Y12 inhibitors as the most frequently used medications in the management of SCAD [36]. The empiric medications involved in treating SCAD include thrombolytics, anticoagulants, antiplatelets, statins, and beta-blockers. However, the use of these medications is under debate and is further elaborated below.

Thrombolytics: Initiating thrombolysis in SCAD patients has been reported in case reports to have either favorable outcomes or dissection extension [37-39]. This has raised concern for the safety and efficacy of thrombolytics and hence has been advised against by the European Society of Cardiology-ACC Association [35].

Antiplatelet and P2Y12 inhibitors: The rationale for antiplatelet agents is justified by a thrombus in the true lumen causing SCAD-related ischemia. Valgimigli et al. recommend dual antiplatelet agents (aspirin and clopidogrel) during the acute phase with lifelong aspirin for conservatively managed patients and 12-month dual antiplatelet therapy for patients managed by stent implants [40]. However, the DISCO clinical study reported a 15% risk of major adverse cardiac events (MACE) on one-year follow-up in patients prescribed with dual antiplatelet regimen compared to single antiplatelet therapy [41].

Anticoagulant agents: Al-Hussaini et al. encourage limiting anticoagulant therapy until revascularization procedures and avoiding long-term use unless clinically indicated by other phenomena such as left ventricular thrombus or embolus [42]. However, Hayes et al. recommend that an anticoagulant regimen using heparin or fondaparinux must be discontinued once SCAD diagnosis is confirmed [5]. As such, the use of anticoagulants is still under debate.

Lipid-lowering agents: The use of lipid-lowering agents can increase the recurrence of SCAD and should only be used as a primary prevention or selectively based on clinical indicators [5,8,33,35]. The mechanism of this is poorly understood but is suspected to be due to the patient population studied, the index of events, and medication use that was started after discharge [5]. The ongoing statin and angiotensin‐converting enzyme inhibitor on symptoms in patients with SCAD (SAFER-SCAD) trial (NCT02008786) aims to address statin and anti-hypertensive efficacy in improving chest pain frequency in SCAD patients.

Beta-blockers and other agents: There is a lack of studies assessing the effect of these agents on SCAD patients. Treating SCAD patients with angiotensin-converting enzyme inhibitors, angiotensin receptor antagonists, mineralocorticoid receptor antagonists, beta-blockers, nitrates, or calcium channel blockers is recommended in the setting of SCAD with left ventricular systolic function impairment [35]. The use of beta-blockers in SCAD survivors showed reduced recurrence and is part of the long-term regimen for SCAD as per current AHA guidelines [5,43]. A large, three-year-prospective study on long-term complications of SCAD survivors depicts a 19.9% risk of MACE, and a SCAD recurrence rate was seen in 10.4% of the patients. Multivariate modeling of these patients expressed increased recurrence risk due to hypertension and a protective effect demonstrated by beta-blocker consumption [43]. The beta-blockers and antiplatelet agents in patients with BA-SCAD (NCT04850417) is an ongoing prospective, randomized clinical trial that aims to achieve the unmet scientific evidence of beta-blockers and antiplatelet therapy for the treatment of SCAD.

Revascularization Modalities

Conservative management: The clinical course of SCAD, aside from medical therapy, is approached mainly by watchful waiting without indulging in invasive measures such as PCI particularly, in cases of hemodynamic stability and mild coronary stenosis [32]. Around 90% of these patients experienced an uneventful inpatient course confirming the favorable treatment response of this approach [1,24]. This evidence is supported by resultant spontaneous angiographic vessel healing in 95% of SCAD lesions seen on repeat angiograms after 30 days from the index event [44]. A retrospective analysis showed an increased deviation towards conservative management by 2019 (89%) when compared prior to 2013 (35%), which determines the positive impact of this strategy [45]. Adlam et al. have published an exhaustive compilation of case series from Mayo Clinic, Canadian, Japanese, and Swiss cohorts describing the percentage of healed cases in conservatively managed patients, which was as high as 76% [35]. Additionally, Wong et al. reported the optimal time to heal as 80 days from diagnosis time, with around 65.8% of lesions healed by 40.5 days of the inciting event [30]. The evidence on recurrent SCAD shows that it does not recur in the same location, which defeats the purpose of preventative revascularization stents [46].

Percutaneous coronary intervention (PCI): While routine revascularization is not recommended, PCI is needed in a notable subset of cases of ongoing symptomatic ischemia, patient instability, vessel occlusion, or high-risk anatomy [5,35,47,48,49]. A retrospective study of 53 SCAD patients showed a lower revascularization success rate but overall better survival in conservative versus PCI strategy than patients with atherosclerotic ST-segment elevation MI (STEMI) [50]. PCI intervention conveys a high rate of periprocedural failure and complications such as secondary iatrogenic dissections or intramural hematoma advancement by the PCI wires in existing feeble coronary structures [48,49,51-53]. Larger sample retrospective studies and meta-analyses conducted have further noted a failure rate of 35-53% with PCI strategy and urgent referral to coronary artery bypass grafting (CABG) in 9-13% of patients. SCAD patients were more prone to emergency CABG when exposed to PCI versus conservative strategy [48,54]. In a case-control study of 158 SCAD survivors, 35% underwent PCI, with two-thirds undergoing stenting and 3% warranting CABG. Additionally, 44% of these patients experienced complications with the majority due to hematoma extension and others due to distal coronary occlusion [55].

Despite these limitations, the focus has diverted to techniques that decompress the false arterial lumen through low caliber scoring/cutting balloon angioplasty that fenestrate the hematoma within the intima and media membrane and reduce compression of the true lumen [56-58]. The Polish Cardiac Society has gathered evidence stating the need to ensure special attention to catheter maneuvers with guidewire entry into the true lumen rather than stent deployment in the false lumen or collateral occlusion due to hematoma expansion. The aim should follow the restoration of TIMI 3 flow with minimally invasive techniques such as plain old balloon angioplasty, longer stents, or focal entry with short stents to seal the entry tear [34,43,44,54]. There is an added shift of interest to utilizing drug-eluting stents (DES), instead of metallic ones, as these patients are of otherwise no atherosclerotic damage [59]. In a recent case with MI and SCAD, PCI using a second-generation DES was successful in healing an aneurysmal false lumen in the LAD with the risk of proximal progression [60]. The use of bioresorbable vascular scaffolds (BVS) is an emerging strategy that is completely devoid of a metal layer with decreased risk of restenosis as was seen in the multicenter prospective study in 15 high-risk SCAD patients that received 34 bioresorbable scaffolds. They were assessed two years later on CCTA, showing no significant restenosis and optimal vessel wall healing [61,62]. BVS can be difficult in navigation and crossability during implantation, but because of the low atherosclerotic nature of the SCAD vessels, this pitfall can be tended to [63]. In three patients with coronary blood flow jeopardy, Macaya et al. demonstrated success via OCT-guided revascularization using bioresorbable scaffolds. The OCT facilitated the decision for BVS, which was favorable as these scaffolds also aid in endothelial restoration and decreased tear-tension on the vessel [64]. A retrospective multicenter study of 238 patients had 108 patients undergoing PCI with DES or bare metal stents where 24 of these patients developed PCI complications. They also observed a downward trend over three years in MACE in DES (26%) compared to bare metal stents (39%) [65]. These data corroborate existing evidence on the risk of bare metal stents in non-SCAD PCI [35,59]. However, further studies are needed for better implications and optimal techniques of revascularization [65,66].

CABG: It is the bailout modality of choice in cases of failed PCI with ongoing ischemia or if left main/multiple vessels are affected, but it is quite challenging as around 30% of acute graft closure was recorded in patients [48]. Positive long-term outcomes have only been reported in a few cases [48,67].

CR Programs

It is important to highlight that all patients who undergo SCAD be consigned to a CR program as per the AHA guidelines, given the association between SCAD recurrence and physical stress [5]. While studies do promote an active lifestyle in these patients and discourage high-intensity competitive sports, other studies have suggested excellent outcomes in those indulging in high-intensity sports [5,68]. Behavioral interventions that target resilience training could be useful, along with screening for psychological stress factors, as they correlate with low quality of life and current increase in prevalence [34]. Baechler et al. identified terms that describe psychosocial burden such as the frustration of misdiagnosis, follow-up, or resources for mental support [69]. Several schemes for psychosocial interventions have also been advocated such as patient education and peer support sessions, counseling, mindfulness practices, and, to a smaller extent, coping mechanisms [39]. As these can significantly affect the quality of life in survivors, it is necessary to address the support program, and the CR program has been found to deliver these notions [36]. Chacin-Suarez et al. recorded responses from SCAD survivors stating that 93.3% were recommended for physical activity on early follow-up visits and around 2.9% were advised against it [70]. Additional recommendations to avoid Valsalva maneuvers, lifting heavy weights, aerobics, and contact sports were also provided. Murphy et al. provided a qualitative [71] and, more recently, a quantitative study [72] analyzing 48 psychosocial and five lifestyle patient implications. They describe the impending effect of lack of information that patients are bound to experience. Therapies apart from the CR program, such as acceptance and commitment therapy and cognitive behavioral therapy, could aid in alleviating the fear and concern of anxiety, depression, and disease recurrence, including overall psychological support [71,72]. The in-depth analysis reveals recommendations for evidence-based programs that enable emotional and educational support, guidelines, and patient-centered regimens to manage daily activities and work-wise flexibility [72]. The prevalence of MACE measured over 25 years was around 24% with a decreased need for intensive care admissions [73]. However, in a recent larger systematic review, MACE was reportedly increased with SCAD recurrence in 31%, ACS in 27.4%, target vessel revascularization in 30%, and others such as repeat stenting, unstable angina, heart failure, and stroke [53]. An additional benefit observed in the dedicated SCAD-CR program was the resultant decreased complaint of chest pain, higher exercise endurance, and decreased incidence of MACE and recurrent SCAD observed on follow-up [74].

Directional trends

The emerging use of coronary angiography and other modalities has assisted in identifying recurrent SCAD, with rates from 8 to 27% [75]. Screening to identify extra coronary vascular abnormalities, such as fibromuscular dysplasia through imaging, has increased over time from 33% in 2013 to 71% in 2018 [45]. This also indicates that dictating diagnoses such as fibromuscular dysplasia from carotid or renal vessels requires skill and is operator-dependent [76]. Additional screening for reproductive planning, along with genetic testing, has been recommended [24]. Secondary prevention strategies to cope with SCAD have been limited as seen by the systematic analysis of the quality of life for these patients [36]. Apart from large-scale systematic analyses and ongoing medical trials, there are multiple patient registries that serve this purpose and can ensure continuous education and research to attain the end goal of effective patient care [45].

## Conclusions

SCAD is a potentially life-threatening diagnosis that requires prompt identification, diagnosis, and management to achieve a favorable prognosis in affected patients. It is most prevalent in middle-aged women but has also been identified in men and postmenopausal women. Coronary angiography remains the gold standard for diagnosis of SCAD, but when diagnostic doubt persists, other imaging modalities aid in diagnoses, including OCT, intravascular ultrasound, and CCTA. SCAD can be managed medically or invasively, to achieve reperfusion and ultimately, a favorable prognosis. Given that data are lacking, there is a scarcity of an exclusive algorithm for the management of SCAD. This article meticulously highlighted the current evidence supporting the different diagnostic modalities and management strategies to provide practical insights to clinicians in the management of SCAD.
